# IRT studies of many groups: the alignment method

**DOI:** 10.3389/fpsyg.2014.00978

**Published:** 2014-09-12

**Authors:** Bengt Muthén, Tihomir Asparouhov

**Affiliations:** MplusLos Angeles, CA, USA

**Keywords:** factor means invariance testing country comparisons, approximate invariance maximum-likelihood, Bayesian inference, invariance testing, maximum likelihood estimation

## Abstract

Asparouhov and Muthén (2014) presented a new method for multiple-group confirmatory factor analysis (CFA), referred to as the alignment method. The alignment method can be used to estimate group-specific factor means and variances without requiring exact measurement invariance. A strength of the method is the ability to conveniently estimate models for many groups, such as with comparisons of countries. This paper focuses on IRT applications of the alignment method. An empirical investigation is made of binary knowledge items administered in two separate surveys of a set of countries. A Monte Carlo study is presented that shows how the quality of the alignment can be assessed.

## 1. Introduction

Asparouhov and Muthén (2014) presented a new method for multiple-group confirmatory factor analysis (CFA), referred to as the alignment method. The alignment method can be used to estimate group-specific factor means and variances without requiring exact measurement invariance. A strength of the method is the ability to conveniently estimate models for many groups, such as with comparisons of countries. The method is a valuable alternative to the currently used multiple-group CFA methods for studying measurement invariance that require multiple manual model adjustments guided by modification indices. Multiple-group CFA is not practical with many groups due to poor model fit of the scalar model and too many large modification indices. In contrast, the alignment method is based on the configural model and essentially automates and greatly simplifies measurement invariance analysis. The method also provides a detailed account of parameter invariance for every model parameter in every group.

This paper focuses on IRT applications of the alignment method. An empirical investigation is made of binary knowledge items administered in two separate surveys of a set of countries. A Monte Carlo study is presented that shows how the quality of the alignment can be assessed. Mplus inputs are provided in the Supplementary Material.

## 2. Multiple-group IRT

Consider the response to item *y* expressed by the two-parameter logit model for individual *i* in group *g*,

(1) $$P(y_{ig}=1|\eta_{ig})=\frac{1}{1+exp[-a_{g}(\eta_{ig}-b_{g})]},$$

where *g* = 1, …, *G* and *G* is the number of groups, *i* = 1, …, *N_g_* where *N_g_* is the number of independent observations in group *g*, and η_*ig*_ is a latent variable, η_*ig*_ ~ *N*(α_*g*_, ψ_*g*_). Using item response theory (IRT) language, *a_g_* is the discrimination parameter and *b_g_* the difficulty parameter. For a recent overview of IRT for psychologists, see e.g., Reise et al. (2013).

Measurement invariance for *a_g_* and *b_g_* (referred to as “item bias” and “DIF” in IRT) has traditionally been concerned with comparing a small number of groups such as with gender or ethnicity using techniques such as likelihood-ratio chi-square testing of one item at a time (see e.g., Thissen et al., 1993). Two common approaches have been discussed (Stark et al., 2006; Lee et al., 2010; Kim and Yoon, 2011):

- Bottom-up: Start with no invariance (configural case), imposing invariance one item at a time.
- Top-down: Start with full invariance (scalar case), freeing invariance one item at a time.

Neither approach is scalable—both are very cumbersome when there are many groups, such as 50 countries (50 × 49/2 = 1225 pairwise comparisons for each item). The correct model may well be far from either of the two starting points, which may lead to the wrong model. Asparouhov and Muthén (2014) proposed a new method referred to as alignment which is suitable for analysis of many groups. The alignment method is based on the idea of starting from the configural model with no invariance and attempting to find as much invariance as possible by letting the factor means and variances vary across groups.

## 3. The alignment method

Asparouhov and Muthén (2014) considers the model for a continuous item *y_ipg_*,

(2) $$y_{ipg}=\nu_{pg}+\lambda_{pg}\eta_{ig}+\varepsilon_{ipg},$$

where *p* = 1, …, *P* and *P* is the number of observed indicator variables, *g* = 1, …, *G* and *G* is the number of groups, *i* = 1, …, *N_g_* where *N_g_* is the number of independent observations in group *g*, η_*ig*_ is a latent variable and we assume that ε_*ipg* ~ *N*(0, θ_*pg*_)_, η_*ig*_ ~ *N*(α_*g*_, ψ_*g*_). This expression is relevant also for binary outcomes when letting the dependent variable in (2) be a continuous latent response variable y^*^_ipg_ underlying the observed binary variable *y*_*ipg*_, where using a threshold parameter τ,

$$y_{ipg}=\{\begin{array}{ll} 0, & \text{ if}y_{ipg}^{*}\leq \tau_{pg}\\ 1, & \text{ if}y_{ipg}^{*}>\tau_{pg} \end{array}$$

and the variance of the residual ε_*ipg*_ is standardized as π^2^/3 in line with the logistic model (with the alternative probit modeling, the residual variance is standardized as one). Using (2), the IRT parameters of (1) are obtained as

(3) $$a_{pg}=\lambda_{pg},$$

(4) $$b_{pg}=\tau_{pg}/\lambda_{pg}.$$

**Figure 1:**
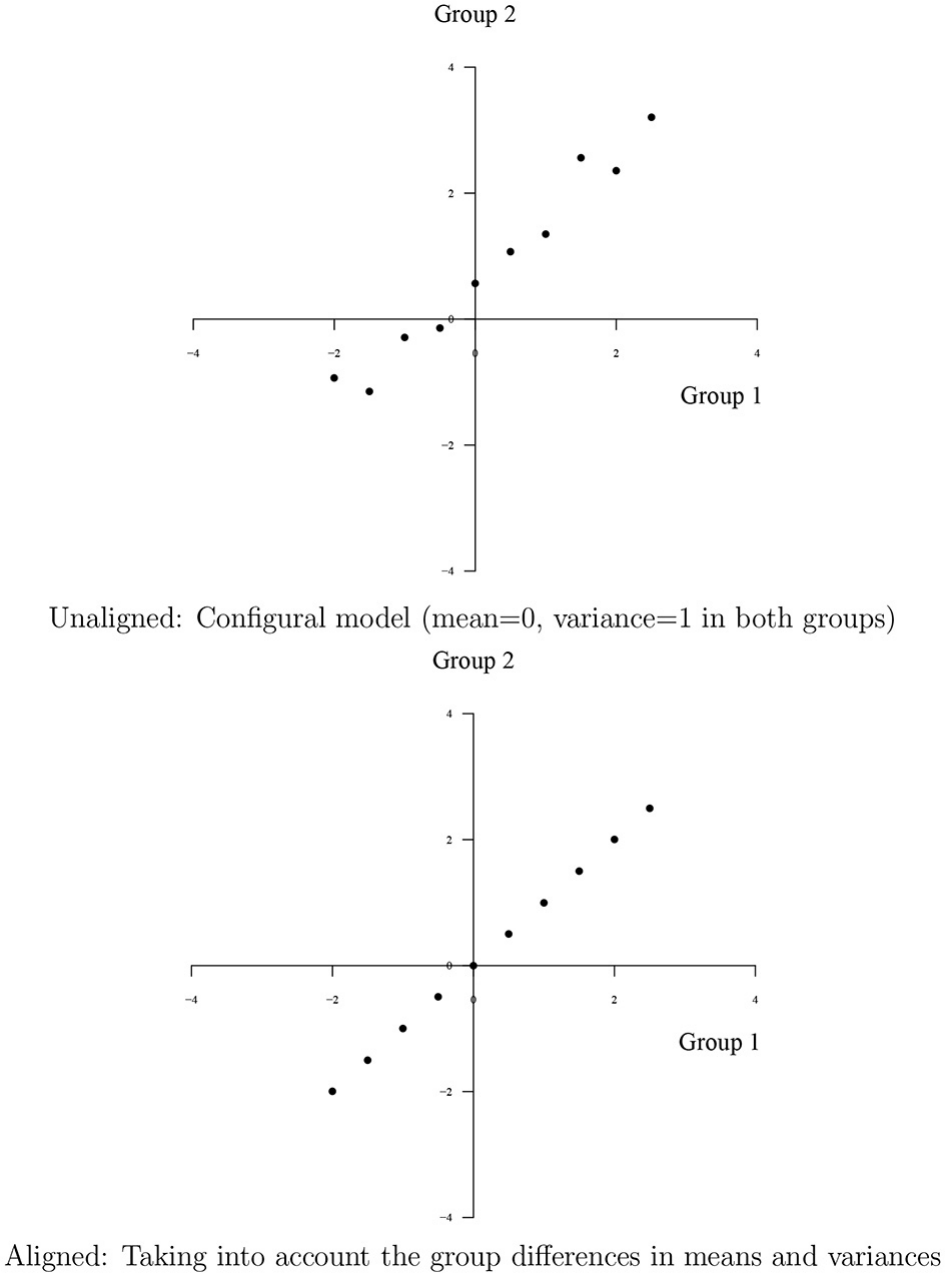
**Unaligned and aligned intercept parameters axes correspond to intercept values for the two groups**. Unaligned: Configural model (mean = 0, variance = 1 in both groups). Aligned: Taking into account the group differences in means and variances.

Asparouhov and Muthén (2014) illustrates the reason for the choice of the term alignment for this new method as in Figure 1 using continuous items. Consider group-invariant intercepts and loadings for 10 items and two groups with factor means 0 and −1 and factor variances 1 and 2. The configural model of the first step of alignment fixes the factor means and variances to 0 and 1, respectively, in both groups. The plot at the top shows the configural intercept parameters which due to group differences in factor means and variances are not equal across the two groups despite the perfect measurement invariance of the original parameters. The plot at the bottom shows the invariance across groups of the original parameters where the correct factor means and variances have been taken into account. Going from the top to the bottom plot, the intercept parameters have been aligned.

### 3.1. The alignment fitting function

Denote the estimates of the configural model by ν_*pg*, 0_ and λ_*pg*, 0_. Asparouhov and Muthén (2014) show that for every set of parameters α_*g*_ and ψ_*g*_ there are intercept and loading parameters ν_*pg*_ and λ_*pg*_ that yield the same likelihood as the configural model. These parameters can be obtained as follows

(5) $$\lambda_{pg,1}=\frac{\lambda_{pg,0}}{\sqrt{\psi_{g}}},$$

(6) $$\nu_{pg,1}=\nu_{pg,0}-\alpha_{g}\frac{\lambda_{pg,0}}{\sqrt{\psi_{g}}}.$$

We want to choose α_*g*_ and ψ_*g*_ so that we minimize the amount of measurement non-invariance. The α_*g*_ and ψ_*g*_ parameters are, however, not identified in the configural model and are fixed to zero and one, respectively for each group. Adding a simplicity function gives the necessary restrictions to identify the model. The simplicity function minimizes with respect to α_*g*_ and ψ_*g*_ the total loss/simplicity function *F* which accumulates the total measurement non-invariance over the items,

(7) $$\begin{array}{l} F=\sum_{p}\sum_{g_{1}<g_{2}}w_{g_{1},g_{2}}f(\lambda_{pg_{1},1}-\lambda_{pg_{2},1})\\ \;+\sum_{p}\sum_{g_{1}<g_{2}}w_{g_{1},g_{2}}f(\nu_{pg_{1},1}-\nu_{pg_{2},1}). \end{array}$$

The function *F* implies that for every pair of groups and every intercept and loading parameter we add to the total loss function the difference between the parameters scaled via the component loss function (CLF) *f*. CLF has been used in EFA analysis, see for example Jennrich (2006) and it is used similarly here. One good choice for the CLF is

$$f(x)=\sqrt{\sqrt{x^{2}+\epsilon }}$$

where ϵ is a small number such as 0.0001. Thus, the total loss function *F* will be minimized at a solution where there are a few large non-invariant measurement parameters and many approximately invariant measurement parameters rather than many medium-sized non-invariant measurement parameters. This is similar to the fact that EFA rotation functions aim for either large or small loadings, but not mid-sized loadings.

The alignment method is carried out using maximum-likelihood estimation of the configural model. In addition to the logit model, probit can also be handled. More than one factor can also be accommodated in which case the alignment is done for each factor. Cross-loadings are not, however, allowed. To handle national surveys, the estimation allows complex survey data with stratification, weights, and clustering, where standard errors are computed using the Huber-White sandwich estimator.

Muthén and Asparouhov (2013a) make a comparison of the alignment method and two-level IRT modeling. In the former approach the groups are viewed as a fixed mode of variation, whereas in the latter approach they are viewed as a random mode of variation. A key advantage of the alignment method is that a specific distributional assumption such as normality of the item parameter distributions across groups is not required. For example, a subset of the groups may show large non-invariance, whereas the remaining groups may show little invariance. Information about which groups contribute to non-invariance is also more readily available with the alignment method.

### 3.2. Alignment quality and degree of non-invariance

In discussing the quality of the alignment results, Asparouhov and Muthén (2014) stated

“The alignment method will always estimate the simplest model with the largest amount of invariance, but if the assumption of approximate measurement invariance is violated the simplest and most invariant model may not be the true model. For example, if data are generated where a minority of the factor indicators have invariant measurement parameters and the majority of the indicators have the same amount of non-invariance, the alignment method will choose the non-invariant indicators as the invariant ones, singling out the other indicators as non-invariant.”

The Asparouhov and Muthén (2014) simulation results show that alignment parameter biases increase with increasing degree of measurement non-invariance, decreasing group sample size, and increasing number of groups. For 60 groups, satisfactory results were obtained with groups sizes of 1000 and at most 20% non-invariant measurement parameters. A key issue is the quality of the ranking of groups based on factor means. Monte Carlo simulations in Muthén and Asparouhov (2013a) focused on the correlation between the population factor means and the estimated alignment factor means computed over groups and averaged over replications. Correlations of at least 0.98 were deemed to produce reliable factor mean rankings. Correlations of this magnitude were seen even in cases with higher than 20% non-invariant measurement parameters. As a rough rule of thumb, a limit of 25% non-invariance may be safe for trustworthy alignment results, while with higher percentages a Monte Carlo simulation study is recommended. Such a study is illustrated below.

## 4. An illustration comparing countries in two cross-sectional surveys

The IEA (International Association for the Evaluation of Educational Achievement) civic knowledge test of 1999 consists of 38 dichotomously scored items. This test, referred to as CIVED, was administered to nearly 90,000 14-year-old students in 28 countries (Torney-Purta et al., 2001; Schultz and Sibberns, 2004). A later survey referred to as ICCS (International Civic and Citizenship Education Study) was carried out in 2009 including 17 link items to make scores comparable to those of 1999 (Schultz et al., 2010). ICCS surveyed over 140,000 eight grade students in 38 countries. 17 countries had comparable national samples and test items and therefore allow comparisons to be made between CIVED achievement and ICCS achievement. Three of these countries had missing data for everyone on at least one of the items at one of the surveys, leaving 14 countries to be compared between the 1999 CIVED and the 2009 ICCS in the current analyses. To further sharpen the comparison, the analyses are restricted to 14-year olds. The IRT alignment analyses to be reported thereby focus on the 17 link items and 29,449 students in 14 countries of CIVED and 10,643 students in 14 countries of ICCS. The 14 countries (country number and country acronym given in parentheses) are: Chile (04; CHL), Colombia (05; COL), Czech Republic (07; CHE), England (09; ENG), Finland (11; FIN), Greece (13; GRC), Italy (16; ITA), Latvia (17; LVA), Norway (19; NOR), Poland (20; POL), Slovak Republic (24; SVK), Slovenia (25; SVN), Sweden (26; SWE), and Switzerland (27; CHE).

Before doing the alignment analysis it is of interest to study measurement invariance using traditional methods, namely comparing the configural, metric, and scalar models (see Muthén and Asparouhov, 2013a). The metric model specifies invariant loadings. The scalar model is of particular interest because it specifies measurement invariance of both thresholds and loadings, a requirement for comparing factor means using traditional methods. Table 1 shows the results for the 1999 CIVED data, the 2009 ICCS data, and the combined data. It is clear that both the metric and the scalar models are rejected by the likelihood-ratio chi-square tests. Part of the reason for this is that the sample sizes are large so there is considerable power to reject invariance. Although criteria such as difference in global fit indices like CFI or RMSEA (Chen, 2007) or detection of local misspecification (Saris et al., 2009) have been proposed to somewhat mitigate this power issue, they are not available with the maximum-likelihood estimation of binary items considered here.

**Table 1:** **Configural, metric, and scalar invariance**.

**INVARIANCE TESTING - CIVED1999 (14 GROUPS)**			
**Model**	**Number of parameters**	**Loglikelihood**	
Configural	489	−343840.898	
Metric	281	−344830.191	
Scalar	73	−354806.259	
**Models compared**	**Chi-square**	**Degrees of freedom**	***P*-value**
Metric against configural	1331.149	208	0.0000
Scalar against configural	13535.800	416	0.0000
Scalar against metric	11375.032	208	0.0000
**INVARIANCE TESTING - ICSS2009 (14 GROUPS)**			
**Model**	**Number of parameters**	**Loglikelihood**	
Configural	489	−126423.673	
Metric	281	−126779.127	
Scalar	73	−130742.955	
**Models compared**	**Chi-square**	**Degrees of freedom**	***P*-value**
Metric against Configural	580.862	208	0.0000
Scalar against Configural	7110.001	416	0.0000
Scalar against Metric	6573.006	208	0.0000
**INVARIANCE TESTING - CIVED1999 AND ICSS2009 (28 GROUPS)**			
**Model**	**Number of parameters**	**Loglikelihood**	
Configural	979	−493498.177	
Metric	547	−494909.372	
Scalar	115	−509271.808	
**Models compared**	**Chi-square**	**Degrees of freedom**	***P*-value**
Metric against configural	2083.617	432	0.0000
Scalar against configural	22223.702	864	0.0000
Scalar against metric	19349.849	432	0.0000

Whatever step-wise non-invariance search method is applied, a large effort is required to find subsets of items that fulfill scalar invariance sufficiently well in different subsets of the groups. The advantage of the alignment method is that metric and scalar invariance are not required. Instead, factor means are made comparable while minimizing measurement non-invariance.

A 14-group alignment analysis of the 17 items is performed for the 14 countries in each of the two surveys, followed by a 28-group alignment analysis of the two surveys jointly. The joint analysis makes it possible to compare factor means and factor variances not only across countries but also across the two surveys. The survey-specific analyses are used to check that the ordering of countries is not largely affected by considering the two surveys together. It was found that the country ordering was almost exactly the same within studies as in the joint 28-group alignment analysis.

The results of the 28-group joint analysis are shown in Tables 2, 3 in factor analysis metric for thresholds and loadings, respectively. The tables indicate which item parameters are non-invariant in which groups by putting groups in parentheses. It is seen that even after alignment many item parameters remain significantly non-invariant in many of the groups. An interesting feature of alignment is that this does not invalidate the alignment method. Thirty three percent of the thresholds and 11% of the loadings are found non-invariant, averaging to 22% non-invariance. Using the 25% rule of thumb mentioned earlier, this implies trustworthy alignment results. To support this conclusion, Monte Carlo simulations reported in Section 5 based on these parameter estimates show that the factor means are well estimated so that a group comparison can be made.

**Table 2:** **Invariance results for aligned threshold parameters for items Y1 to Y17 (numbers in parentheses refer to countries that show significant non-invariance for the parameter)**.

Y1	**(104)** 105 **(107) (109)** 111 113 116 117 119 120 124 125 **(126)** 127 **(204)**
	205 **(207)** 209 **(211)** 213 216 217 219 **(220)** 224 225 226 227
Y2	**(104) (105)** 107 **(109)** 111 **(113) (116)** 117 **(119)** 120 **(124) (125) (126) (127)**
	**(204) (205)** 207 **(209)** 211 **(213) (216)** 217 **(219)** 220 224 225 **(226) (227)**
Y3	**(104) (105)** 107 109 111 113 **(116)** 117 119 120 124 125 **(126)** 127 **(204)**
	**(205) (207)** 209 211 213 216 **(217)** 219 220 224 225 226 227
Y4	104 **(105)** 107 **(109)** 111 113 **(116) (117)** 119 120 124 125 126 127 204 205
	207 209 211 213 216 217 219 220 224 225 226 227
Y5	104 105 107 109 111 113 116 117 **(119)** 120 124 125 **(126)** 127 **(204) (205)**
	207 209 **(211) (213)** 216 **(217) (219)** 220 224 225 **(226)** 227
Y6	**(104) (105)** 107 **(109)** 111 **(113) (116)** 117 119 120 **(124)** 125 126 **(127)**
	204 205 207 209 211 **(213) (216)** 217 219 220 **(224)** 225 226 227
Y7	**(104) (105)** 107 109 111 113 116 117 119 120 124 125 126 **(127)** 204 205
	**(207)** 209 211 213 216 **(217)** 219 220 224 225 226 227
Y8	**(104)** 105 107 109 111 113 116 117 119 **(120)** 124 **(125) (126)** 127 **(204)**
	205 **(207)** 209 211 213 216 217 219 **(220) (224)** 225 226 **(227)**
Y9	**(104) (105) (107) (109) (111) (113)** 116 **(117) (119)** 120 **(124)** 125 **(126)**
	**(127) (204) (205) (207)** 209 **(211)** 213 216 **(217) (219)** 220 **(224)** 225 **(226)** 227
Y10	104 105 107 **(109) (111) (113)** 116 117 **(119)** 120 124 125 **(126)** 127 204
	205 **(207)** 209 **(211)** 213 216 217 219 220 224 225 **(226)** 227
Y11	104 **(105)** 107 109 111 113 116 **(117)** 119 **(120)** 124 125 126 127 204 **(205)**
	**(207) (209)** 211 213 216 217 219 220 224 225 **(226)** 227
Y12	**(104) (105) (107) (109) (111)** 113 **(116)** 117 119 **(120)** 124 **(125)** 126 **(127)**
	204 205 207 **(209)** 211 213 216 217 219 220 224 225 226 227
Y13	**(104) (105)** 107 **(109)** 111 **(113)** 116 **(117)** 119 120 124 **(125)** 126 127 204
	205 **(207)** 209 211 213 216 217 219 220 224 225 226 227
Y14	104 **(105)** 107 **(109)** 111 **(113)** 116 117 **(119)** 120 **(124) (125)** 126 127 204
	**(205)** 207 209 211 **(213)** 216 217 219 220 224 225 226 227
Y15	104 105 **(107) (109) (111)** 113 116 **(117) (119)** 120 124 **(125)** 126 **(127)**
	204 **(205)** 207 **(209)** 211 213 216 **(217) (219)** 220 224 225 **(226)** 227
Y16	104 105 107 109 111 **(113)** 116 **(117)** 119 120 124 125 **(126) (127) (204)**
	205 207 209 211 213 216 **(217)** 219 220 224 225 **(226)** 227
Y17	**(104) (105)** 107 109 111 113 **(116)** 117 119 120 124 **(125)** 126 127 204 205
	**(207) (209)** 211 213 216 217 **(219)** 220 **(224)** 225 226 227

*The group values correspond to the country coding, where a first digit 1 refers to the CIVED survey, a first digit 2 refers to the ICCS survey, and the next two digits correspond to the country codes given in the text*.

**Table 3:** **Invariance results for aligned loadings for items Y1 to Y17 (numbers in parentheses refer to countries that show significant non-invariance for the parameter)**.

Y1	104 105 107 **(109)** 111 113 **(116)** 117 **(119)** 120 124 125 126 127 204 205
	207 209 211 213 216 217 219 220 224 225 226 227
Y2	104 **(105)** 107 109 111 113 **(116)** 117 119 120 124 125 126 **(127)** 204 205
	207 209 211 213 216 217 219 220 224 225 226 227
Y3	104 105 107 109 111 113 116 117 **(119) (120) (124) (125)** 126 127 204 205
	207 209 211 213 216 217 219 220 224 225 226 227
Y4	104 105 107 109 111 113 116 117 119 120 124 125 126 127 204 205 207 209
	211 213 216 217 219 220 224 225 226 227
Y5	104 105 107 109 111 113 116 117 **(119)** 120 124 125 **(126)** 127 204 205 207
	209 211 213 216 217 219 220 224 225 226 227
Y6	104 105 107 109 111 **(113)** 116 117 **(119) (120)** 124 125 126 127 204 205
	207 209 **(211)** 213 216 217 219 220 224 225 226 227
Y7	104 105 107 109 **(111) (113)** 116 117 119 120 124 125 126 127 204 205 207
	209 211 **(213) (216)** 217 219 220 224 225 226 227
Y8	104 105 **(107)** 109 **(111)** 113 116 117 **(119)** 120 124 125 126 **(127)** 204 205
	**(207)** 209 211 213 216 217 219 220 224 225 226 **(227)**
Y9	104 105 107 109 **(111) (113)** 116 117 119 120 124 **(125)** 126 127 **(204)** 205
	**(207)** 209 **(211)** 213 216 217 219 220 224 225 226 227
Y10	104 105 107 109 111 113 116 117 119 120 124 125 126 127 204 **(205)** 207
	209 211 213 216 217 219 220 224 225 226 227
Y11	104 105 107 **(109)** 111 113 116 117 119 120 124 125 126 127 204 **(205)** 207
	209 211 213 216 217 219 220 224 225 226 227
Y12	**(104)** 105 107 **(109)** 111 113 **(116)** 117 119 120 124 125 126 127 **(204)** 205
	207 209 211 213 216 217 219 220 224 225 226 227
Y13	104 105 107 109 111 113 116 117 119 120 124 125 126 127 204 205 207 209
	211 213 216 217 219 220 224 225 226 227
Y14	104 105 107 109 111 **(113)** 116 117 119 120 124 125 126 127 204 205 207
	209 211 213 216 217 219 220 224 225 **(226)** 227
Y15	104 105 **(107) (109) (111) (113)** 116 117 119 **(120)** 124 125 126 127 204
	205 **(207) (209)** 211 213 216 217 219 220 224 225 226 227
Y16	104 105 107 109 111 113 116 **(117)** 119 120 124 **(125) (126)** 127 204 205
	207 209 211 213 216 217 219 220 224 225 **(226)** 227
Y17	104 105 107 109 111 113 116 117 119 120 124 125 126 127 204 205 207 209
	211 213 216 217 219 220 224 225 226 227

*The group values correspond to the country coding, where a first digit 1 refers to the CIVED survey, a first digit 2 refers to the ICCS survey, and the next two digits correspond to the country codes given in the text*.

The results in Tables 2, 3 can be augmented by the contributions each item and each group makes to the simplicity function (7). It is of interest to see which items and which groups contribute the most and the least to the non-invariance as quantified by this function. The results can be studied for thresholds and loadings separately or together for an item. It is found that the two least invariant items are items 2 and 9 and the most invariant item is item 4. This largely agrees with the significance findings in Tables 2, 3. Further inspection of these items is therefore warranted. None of the 28 groups stands out as contributing substantially more to the simplicity function, while three groups stand out as contributing the least to the simplicity function: 225 (Slovenia at the second survey), 213 (Greece at the second survey), and 219 (Norway at the second survey).

**Figure 2:**
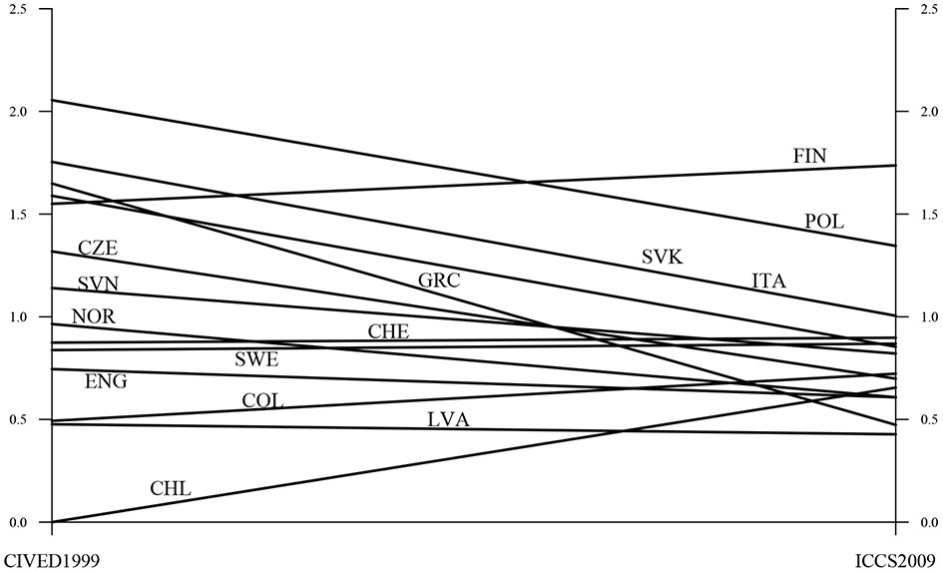
**Factor means for CIVED 1999 and ICCS 2009**.

The aligned factor means are shown in Table 4. The tables also show results of testing for significant factor mean differences between the countries. Figure 2 gives a graphic representation of factor means at the two surveys. It is seen that a majority of the countries decrease in achievement over the 10 years. Exceptions are Finland, the Czech Republic, Sweden, Columbia, and Chile. The variation in the factor means is also diminished such that fewer countries are at the high end on the factor in 2009 as compared to 1999. It is of interest for test developers to investigate if the causes of these features are partly due to testing artifacts. Such an investigation may include studying differences in item order in the testing booklets, different missing data patterns, and different motivation among the students.

**Table 4:** **Factor means**.

**Ranking**	**Group value**	**Factor mean**	**Groups with significantly smaller factor mean**
1	120	2.055	113 124 111 220 107 125 216 119 227 127
			226 224 126 225 109 205 207 204 209 219
			105 117 213 217 104
2	16	1.754	220 107 125 216 119 227 127 226 224 126
			225 109 205 207 204 209 219 105 117 213
			217 104
3	211	1.737	220 107 125 216 119 227 127 226 224 126
			225 109 205 207 204 209 219 105 117 213
			217 104
4	113	1.649	220 107 125 216 119 227 127 226 224 126
			225 109 205 207 204 209 219 105 117 213
			217 104
5	124	1.589	107 125 216 119 227 127 226 224 126 225
			109 205 207 204 209 219 105 117 213 217
			104
6	111	1.550	107 125 216 119 227 127 226 224 126 225
			109 205 207 204 209 219 105 117 213 217
			104
7	220	1.345	216 119 227 127 226 224 126 225 109 205
			207 204 209 219 105 117 213 217 104
8	107	1.318	216 119 227 127 226 224 126 225 109 205
			207 204 209 219 105 117 213 217 104
9	125	1.140	127 226 224 126 225 109 205 207 204 209
			219 105 117 213 217 104
10	216	1.005	109 205 207 204 209 219 105 117 213 217
			104
11	119	0.965	109 205 207 204 209 219 105 117 213 217
			104
12	227	0.898	209 219 105 117 213 217 104
13	127	0.874	209 219 105 117 213 217 104
14	226	0.869	204 209 219 105 117 213 217 104
15	224	0.854	209 105 117 213 217 104
16	126	0.838	209 219 105 117 213 217 104
17	225	0.821	105 117 217 104
18	109	0.745	105 117 217 104
19	205	0.723	217 104
20	207	0.699	117 217 104
21	204	0.655	217 104
22	209	0.608	104
23	219	0.608	104
24	105	0.493	104
25	117	0.477	104
26	213	0.474	104
27	217	0.428	104
28	104	0.000	

*The group values correspond to the country coding, where a first digit 1 refers to the CIVED survey, a first digit 2 refers to the ICCS survey, and the next two digits correspond to the country codes given in the text*.

## 5. Monte Carlo investigation

A useful augmentation of the alignment analysis is to carry out a Monte Carlo simulation study to check how well the factor means are captured. Studies may show a large degree of measurement non-invariance, that is, many measurement parameters show large non-invariance in many groups. The concern may then be that the factor means are not well enough estimated to afford a trustworthy comparison across the groups.

The Monte Carlo study can be done using the same features as in the real-data analysis. The features include the degree of measurement non-invariance, the group-varying factor means and variances, the number of items, the number of groups, and the sample sizes in the groups. Such a Monte Carlo analysis is easily carried out using Mplus. The estimated parameters in the real-data alignment analysis can be saved and used for data generation. A large number of replications (random samples of observations) is used. Summary statistics are provided that include the correlation between the generated and estimated factor means for the countries. A near-perfect correlation is required for the ordering of groups with respect to the factors to be trustworthy. Muthén and Asparouhov (2013a) observed that a correlation of at least 0.98 is needed. For the current 28-group analysis a correlation of 0.996 is observed suggesting excellent alignment despite the non-invariance. The parameter values are also well recovered. Mplus input excerpts for both the real-data and Monte Carlo analyses are shown in the Supplementary Material.

## 6. Conclusions

The alignment method provides a convenient and powerful method to study IRT modeling in many groups. In recent research 92 groups has proved feasible (Munck et al., 2014). With country comparison it is expected that a large degree of non-invariance is present due to cultural and other country differences. Existing methods are simply not practical for handling such complexity. In the current paper maximum-likelihood estimation was used but Bayesian analysis is also available as discussed in Muthén and Asparouhov (2013a). Bayesian analysis also makes it possible to relax the assumptions of the configural IRT model, for example by allowing certain residual correlations among the items. Bayesian analysis also makes it possible to base the alignment on a model with approximate measurement invariance as discussed in Muthén and Asparouhov (2013b).

Future developments of the alignment method for IRT applications include allowing for different booklets administered to different student groups, adding covariates to the alignment method, and the possibility to create plausible values of the factor scores for secondary analyses. These developments should make IRT alignment an even more valuable addition to the IRT methods arsenal.

### Conflict of interest statement

The authors are developers of the Mplus software used in the paper. The authors declare that the research was conducted in the absence of any commercial or financial relationships that could be construed as a potential conflict of interest.
