# Supplementary material for: IRT studies of many groups: the alignment method
Source: Front Psychol. 2014 Sep 12;5:978. doi: 10.3389/fpsyg.2014.00978 (PMC4162377; doi:10.3389/fpsyg.2014.00978)
Supplement: Supplementary file 1 [file Table1.PDF]

List of Tables

1 Configural, Metric, and Scalar Invariance 24
2 Invariance results for aligned threshold parameters for items Y1 to Y17 (numbers in parentheses refer to countries that show significant non-invariance for the parameter) 25
3 Invariance results for aligned loadings for items Y1 to Y17 (numbers in parentheses refer to countries that show significant non-invariance for the parameter) 26
4 Factor means 27
5 Factor means continued 28
6 Mplus input for 28-group alignment analysis 29
7 Mplus input excerpts for Monte Carlo simulation 30
8 Mplus input excerpts for Monte Carlo simulation, continued . . . 31

Table 1: Configural, Metric, and Scalar Invariance

Invariance Testing - CIVED1999 (14 groups)

Model	Number of Parameters	Loglikelihood
Configural	489	-343840.898
Metric	281	-344830.191
Scalar	73	-354806.259

Models Compared	Chi-Square	Degrees of Freedom	P-value
Metric against Configural	1331.149	208	0.0000
Scalar against Configural	13535.800	416	0.0000
Scalar against Metric	11375.032	208	0.0000

Invariance Testing - ICSS2009 (14 groups)

Model	Number of Parameters	Loglikelihood
Configural	489	-126423.673
Metric	281	-126779.127
Scalar	73	-130742.955

Models Compared	Chi-Square	Degrees of Freedom	P-value
Metric against Configural	580.862	208	0.0000
Scalar against Configural	7110.001	416	0.0000
Scalar against Metric	6573.006	208	0.0000

Invariance Testing - CIVED1999 and ICSS2009 (28 groups)

Model	Number of Parameters	Loglikelihood
Configural	979	-493498.177
Metric	547	-494909.372
Scalar	115	-509271.808

Models Compared	Chi-Square	Degrees of Freedom	P-value
Metric against Configural	2083.617	432	0.0000
Scalar against Configural	22223.702	864	0.0000
Scalar against Metric	19349.849	432	0.0000

Table 2: Invariance results for aligned threshold parameters for items Y1 to Y17 (numbers in parentheses refer to countries that show significant non-invariance for the parameter)

Y1	**(104)** 105 **(107)** **(109)** 111 113 116 117 119 120 124 125 **(126)** 127 **(204)** 205 **(207)** 209 **(211)** 213 216 217 219 **(220)** 224 225 226 227
Y2	**(104)** **(105)** 107 **(109)** 111 **(113)** **(116)** 117 **(119)** 120 **(124)** **(125)** **(126)** **(127)** **(204)** **(205)** 207 **(209)** 211 **(213)** **(216)** 217 **(219)** 220 224 225 **(226)** **(227)**
Y3	**(104)** **(105)** 107 109 111 113 **(116)** 117 119 120 124 125 **(126)** 127 **(204)** **(205)** **(207)** 209 211 213 216 **(217)** 219 220 224 225 226 227
Y4	104 **(105)** 107 **(109)** 111 113 **(116)** **(117)** 119 120 124 125 126 127 204 205 207 209 211 213 216 217 219 220 224 225 226 227
Y5	104 105 107 109 111 113 116 117 **(119)** 120 124 125 **(126)** 127 **(204)** **(205)** 207 209 **(211)** **(213)** 216 **(217)** **(219)** 220 224 225 **(226)** 227
Y6	**(104)** **(105)** 107 **(109)** 111 **(113)** **(116)** 117 119 120 **(124)** 125 126 **(127)** 204 205 207 209 211 **(213)** **(216)** 217 219 220 **(224)** 225 226 227
Y7	**(104)** **(105)** 107 109 111 113 116 117 119 120 124 125 126 **(127)** 204 205 **(207)** 209 211 213 216 **(217)** 219 220 224 225 226 227
Y8	**(104)** 105 107 109 111 113 116 117 119 **(120)** 124 **(125)** **(126)** 127 **(204)** 205 **(207)** 209 211 213 216 217 219 **(220)** **(224)** 225 226 **(227)**
Y9	**(104)** **(105)** **(107)** **(109)** **(111)** **(113)** 116 **(117)** **(119)** 120 **(124)** 125 **(126)** **(127)** **(204)** **(205)** **(207)** 209 **(211)** 213 216 **(217)** **(219)** 220 **(224)** 225 **(226)** 227
Y10	104 105 107 **(109)** **(111)** **(113)** 116 117 **(119)** 120 124 125 **(126)** 127 204 205 **(207)** 209 **(211)** 213 216 217 219 220 224 225 **(226)** 227
Y11	104 **(105)** 107 109 111 113 116 **(117)** 119 **(120)** 124 125 126 127 204 **(205)** **(207)** **(209)** 211 213 216 217 219 220 224 225 **(226)** 227
Y12	**(104)** **(105)** **(107)** **(109)** **(111)** 113 **(116)** 117 119 **(120)** 124 **(125)** 126 **(127)** 204 205 207 **(209)** 211 213 216 217 219 220 224 225 226 227
Y13	**(104)** **(105)** 107 **(109)** 111 **(113)** 116 **(117)** 119 120 124 **(125)** 126 127 204 205 **(207)** 209 211 213 216 217 219 220 224 225 226 227
Y14	104 **(105)** 107 **(109)** 111 **(113)** 116 117 **(119)** 120 **(124)** **(125)** 126 127 204 **(205)** 207 209 211 **(213)** 216 217 219 220 224 225 226 227
Y15	104 105 **(107)** **(109)** **(111)** 113 116 **(117)** **(119)** 120 124 **(125)** 126 **(127)** 204 **(205)** 207 **(209)** 211 213 216 **(217)** **(219)** 220 224 225 **(226)** 227
Y16	104 105 107 109 111 **(113)** 116 **(117)** 119 120 124 125 **(126)** **(127)** **(204)** 205 207 209 211 213 216 **(217)** 219 220 224 225 **(226)** 227
Y17	**(104)** **(105)** 107 109 111 113 **(116)** 117 119 120 124 **(125)** 126 127 204 205 **(207)** **(209)** 211 213 216 217 **(219)** 220 **(224)** 225 226 227

The group values correspond to the country coding, where a first digit 1 refers to the CIVED survey, a first digit 2 refers to the ICCS survey, and the next two digits correspond to the country codes given in the text.

Table 3: Invariance results for aligned loadings for items Y1 to Y17 (numbers in parentheses refer to countries that show significant non-invariance for the parameter)

Y1	104 105 107 **(109)** 111 113 **(116)** 117 **(119)** 120 124 125 126 127 204 205 207 209 211 213 216 217 219 220 224 225 226 227
Y2	104 **(105)** 107 109 111 113 **(116)** 117 119 120 124 125 126 **(127)** 204 205 207 209 211 213 216 217 219 220 224 225 226 227
Y3	104 105 107 109 111 113 116 117 **(119)** **(120)** **(124)** **(125)** 126 127 204 205 207 209 211 213 216 217 219 220 224 225 226 227
Y4	104 105 107 109 111 113 116 117 119 120 124 125 126 127 204 205 207 209 211 213 216 217 219 220 224 225 226 227
Y5	104 105 107 109 111 113 116 117 **(119)** 120 124 125 **(126)** 127 204 205 207 209 211 213 216 217 219 220 224 225 226 227
Y6	104 105 107 109 111 **(113)** 116 117 **(119)** **(120)** 124 125 126 127 204 205 207 209 **(211)** 213 216 217 219 220 224 225 226 227
Y7	104 105 107 109 **(111)** **(113)** 116 117 119 120 124 125 126 127 204 205 207 209 211 **(213)** **(216)** 217 219 220 224 225 226 227
Y8	104 105 **(107)** 109 **(111)** 113 116 117 **(119)** 120 124 125 126 **(127)** 204 205 **(207)** 209 211 213 216 217 219 220 224 225 226 **(227)**
Y9	104 105 107 109 **(111)** **(113)** 116 117 119 120 124 **(125)** 126 127 **(204)** 205 **(207)** 209 **(211)** 213 216 217 219 220 224 225 226 227
Y10	104 105 107 109 111 113 116 117 119 120 124 125 126 127 204 **(205)** 207 209 211 213 216 217 219 220 224 225 226 227
Y11	104 105 107 **(109)** 111 113 116 117 119 120 124 125 126 127 204 **(205)** 207 209 211 213 216 217 219 220 224 225 226 227
Y12	**(104)** 105 107 **(109)** 111 113 **(116)** 117 119 120 124 125 126 127 **(204)** 205 207 209 211 213 216 217 219 220 224 225 226 227
Y13	104 105 107 109 111 113 116 117 119 120 124 125 126 127 204 205 207 209 211 213 216 217 219 220 224 225 226 227
Y14	104 105 107 109 111 **(113)** 116 117 119 120 124 125 126 127 204 205 207 209 211 213 216 217 219 220 224 225 **(226)** 227
Y15	104 105 **(107)** **(109)** **(111)** **(113)** 116 117 119 **(120)** 124 125 126 127 204 205 **(207)** **(209)** 211 213 216 217 219 220 224 225 226 227
Y16	104 105 107 109 111 113 116 **(117)** 119 120 124 **(125)** **(126)** 127 204 205 207 209 211 213 216 217 219 220 224 225 **(226)** 227
Y17	104 105 107 109 111 113 116 117 119 120 124 125 126 127 204 205 207 209 211 213 216 217 219 220 224 225 226 227

The group values correspond to the country coding, where a first digit 1 refers to the CIVED survey, a first digit 2 refers to the ICCS survey, and the next two digits correspond to the country codes given in the text.

Table 4: Factor means

Ranking	Group Value	Factor Mean	Groups with significantly smaller factor mean
1	120	2.055	113 124 111 220 107 125 216 119 227 127 226 224 126 225 109 205 207 204 209 219 105 117 213 217 104
2	16	1.754	220 107 125 216 119 227 127 226 224 126 225 109 205 207 204 209 219 105 117 213 217 104
3	211	1.737	220 107 125 216 119 227 127 226 224 126 225 109 205 207 204 209 219 105 117 213 217 104
4	113	1.649	220 107 125 216 119 227 127 226 224 126 225 109 205 207 204 209 219 105 117 213 217 104
5	124	1.589	107 125 216 119 227 127 226 224 126 225 109 205 207 204 209 219 105 117 213 217 104
6	111	1.550	107 125 216 119 227 127 226 224 126 225 109 205 207 204 209 219 105 117 213 217 104
7	220	1.345	216 119 227 127 226 224 126 225 109 205 207 204 209 219 105 117 213 217 104
8	107	1.318	216 119 227 127 226 224 126 225 109 205 207 204 209 219 105 117 213 217 104
9	125	1.140	127 226 224 126 225 109 205 207 204 209 219 105 117 213 217 104
10	216	1.005	109 205 207 204 209 219 105 117 213 217 104
11	119	0.965	109 205 207 204 209 219 105 117 213 217 104

The group values correspond to the country coding, where a first digit 1 refers to the CIVED survey, a first digit 2 refers to the ICCS survey, and the next two digits correspond to the country codes given in the text.

Table 5: Factor means continued

Ranking	Group Value	Factor Mean	Groups with significantly smaller factor mean
12	227	0.898	209 219 105 117 213 217 104
13	127	0.874	209 219 105 117 213 217 104
14	226	0.869	204 209 219 105 117 213 217 104
15	224	0.854	209 105 117 213 217 104
16	126	0.838	209 219 105 117 213 217 104
17	225	0.821	105 117 217 104
18	109	0.745	105 117 217 104
19	205	0.723	217 104
20	207	0.699	117 217 104
21	204	0.655	217 104
22	209	0.608	104
23	219	0.608	104
24	105	0.493	104
25	117	0.477	104
26	213	0.474	104
27	217	0.428	104
28	104	0.000	

The group values correspond to the country coding, where a first digit 1 refers to the CIVED survey, a first digit 2 refers to the ICCS survey, and the next two digits correspond to the country codes given in the text.

Table 6: Mplus input for 28-group alignment analysis

TITLE:	MDMI_IEA_CIVIC_TREND_scc34_A4_01
	Grouping scc34: study_1_2 '1'=CIVED99,'2'=ICCS09
	cc is sequential numbering of CIVED99&ICCS09 countries
	N=84674; N(CIVED99)= 59103; N(ICCS99)=25571
	N(strata)=342 N(schools) = 5255
	140121_initial run Alignment method;
DATA:	FILE = MDMI_IEAC_TREND_scc_34.dat;
VARIABLE:	MISSING = ALL (-9);
	NAMES = study_1_2 co_iso3 co_seq2 gr_scc stratum7
	stud15 school10 housewgt f_14y age_3 age grade_8_9
	gender_0_1 lang_0_1 nborn_0_1
	!ci1_121_0_1 ci2_101_0_1 ci3_113_0_1 ci4_115_0_1
	!ci5_106_0_1 ci6_108_0_1 ci7_119_0_1 ci8_120_0_1
	!ci9_127_0_1 ci10_229_0_1 ci11_232_0_1 ci12_204_0_1 ci13_109_0_1
	!ci14_110_0_1 ci15_128_0_1 ci16_130_0_1 ci17_237_0_1;
	y1-y17;
	! Knownclass is used to represent the groups
	CLASSES = c(28);
	KNOWNCLASS = c (gr_scc = 104 105 107 109 111 113 116
	117 119 120 124 125 126 127 204 205 207 209 211 213 216
	217 219 220 224 225 226 227);
	STRATIFICATION = stratum7;
	WEIGHT = housewgt;
	CLUSTER = school10;
	USEVARIABLES = y1-y17;
	CATEGORICAL = y1-y17;
	USEOBSERVATIONS = f_14y eq 1;
ANALYSIS:	TYPE = COMPLEX MIXTURE;
	ALIGNMENT = FIXED(104);
	PROCESSORS = 8;
	ALGORITHM = INTEGRATION;
MODEL:	%OVERALL%
	f BY y1-y17;
OUTPUT:	TECH8 ALIGN SVALUES;
SAVEDATA:	RANKINGS = ranktable.csv;

Table 7: Mplus input excerpts for Monte Carlo simulation

TITLE:	
MONTECARLO:	NAMES = y1-y17;
	NGROUPS = 28;
	NOBSERVATIONS = 28(2000);
	NREPS = 50;
	GENERATE = y1-y17(1);
	CATEGORICAL = y1-y17;
ANALYSIS:	TYPE =MIXTURE;
	ESTIMATOR = ML;
	ALIGNMENT = FIXED(1);
	PROCESSORS = 8;
	ALGORITHM = INTEGRATION;

Table 8: Mplus input excerpts for Monte Carlo simulation, continued

MODEL POPULATION:	%OVERALL%
	f BY y1-y17*1;
	%g#1%
	f BY y1*0.72003;
	f BY y2*0.81696;
	f BY y3*1.15023;
	f BY y4*1.03386;
	f BY y5*1.24624;
	f BY y6*0.56078;
	f BY y7*1.02838;
	f BY y8*1.05826;
	f BY y9*0.68327;
	f BY y10*0.75416;
	f BY y11*0.89518;
	f BY y12*0.60922;
	f BY y13*0.82176;
	f BY y14*0.86823;
	f BY y15*1.16581;
	f BY y16*2.07436;
	f BY y17*1.20428;
	[f*0];
	[y1$1*0.09646];
	[y2$1*0.07228];
	[y3$1*0.85295];
	[y4$1*0.67134];
	[y5$1*-0.78792];
	[y6$1*-0.49738];
	[y7$1*-0.64681];
	[y8$1*-2.35594];
	[y9$1*0.04811];
	[y10$1*0.57027];
	[y11$1*-0.29527];
	[y12$1*-0.50908];
	[y13$1*-0.38591];
	[y14$1*0.30056];
	[y15$1*-0.07502];
	[y16$1*-0.06999];
	[y17$1*-0.17765];
	f*1;
	%g#2%
